# Comparison between quantitative cardiac magnetic resonance perfusion imaging and [^15^O]H_2_O positron emission tomography

**DOI:** 10.1007/s00259-019-04641-9

**Published:** 2019-12-10

**Authors:** Henk Everaars, Pepijn A. van Diemen, Michiel J. Bom, Stefan P. Schumacher, Ruben W. de Winter, Peter M. van de Ven, Pieter G. Raijmakers, Adriaan A. Lammertsma, Mark B. M. Hofman, Rob J. van der Geest, Marco J. Götte, Albert C. van Rossum, Robin Nijveldt, Ibrahim Danad, Roel S. Driessen, Paul Knaapen

**Affiliations:** 1 Department of Cardiology, Amsterdam University Medical Centers, Vrije Universiteit, Amsterdam, the Netherlands; 2 Department of Epidemiology and Biostatistics, Amsterdam University Medical Centers, Vrije Universiteit, Amsterdam, the Netherlands; 3 Department of Radiology and Nuclear Medicine, Amsterdam University Medical Centers, Vrije Universiteit, Amsterdam, the Netherlands; 4 Department of Radiology, Leiden University Medical Centers, Leiden, the Netherlands; 5 Department of Cardiology, Radboud University Medical Center, Nijmegen, the Netherlands

**Keywords:** Cardiovascular magnetic resonance, Positron emission tomography, Quantitative myocardial perfusion, Myocardial blood flow, Myocardial flow reserve

## Abstract

**Purpose:**

To compare cardiac magnetic resonance imaging (CMR) with [^15^O]H_2_O positron emission tomography (PET) for quantification of absolute myocardial blood flow (MBF) and myocardial flow reserve (MFR) in patients with coronary artery disease (CAD).

**Methods:**

Fifty-nine patients with stable CAD underwent CMR and [^15^O]H_2_O PET. The CMR imaging protocol included late gadolinium enhancement to rule out presence of scar tissue and perfusion imaging using a dual sequence, single bolus technique. Absolute MBF was determined for the three main vascular territories at rest and during vasodilator stress.

**Results:**

CMR measurements of regional stress MBF and MFR showed only moderate correlation to those obtained using PET (*r* = 0.39; *P* < 0.001 for stress MBF and *r* = 0.36; *P* < 0.001 for MFR). Bland-Altman analysis revealed a significant bias of 0.2 ± 1.0 mL/min/g for stress MBF and − 0.5 ± 1.2 for MFR. CMR-derived stress MBF and MFR demonstrated area under the curves of respectively 0.72 (95% CI: 0.65 to 0.79) and 0.76 (95% CI: 0.69 to 0.83) and had optimal cutoff values of 2.35 mL/min/g and 2.25 for detecting abnormal myocardial perfusion, defined as [^15^O]H_2_O PET-derived stress MBF ≤ 2.3 mL/min/g and MFR ≤ 2.5. Using these cutoff values, CMR and PET were concordant in 137 (77%) vascular territories for stress MBF and 135 (80%) vascular territories for MFR.

**Conclusion:**

CMR measurements of stress MBF and MFR showed modest agreement to those obtained with [^15^O]H_2_O PET. Nevertheless, stress MBF and MFR were concordant between CMR and [^15^O]H_2_O PET in 77% and 80% of vascular territories, respectively.

## Introduction

Cardiac magnetic resonance imaging (CMR) allows for the noninvasive assessment of myocardial perfusion in patients with suspected coronary artery disease (CAD), and its utilization for this task is recommended by contemporary guidelines [1]. In contrast to cardiac radionuclide modalities such as single-photon emission computed tomography and positron emission tomography (PET), considered the mainstay for the noninvasive evaluation of myocardial perfusion, CMR has superior spatial resolution and does not involve exposure to ionizing radiation. Although CMR perfusion images are predominantly assessed through visual analysis in clinical practice, quantification of absolute myocardial blood flow (MBF) and myocardial flow reserve (MFR) using CMR has gained increased interest. Quantification holds several advantages over a visual read. It is less dependent on the skill and experience of the observer and aids in identifying patients at risk for future cardiac events [2]. Most importantly, quantification may have incremental diagnostic value, particularly in the unraveling of homogenously diminished perfusion due to triple vessel or left main disease and subtle regional ischemia that goes undetected in a visual read. Indeed, previous studies have shown the need for quantitative CMR perfusion for improving detection and management of CAD [3–5]. Absolute quantification of myocardial perfusion with CMR has been validated ex vivo against microspheres [6, 7]. In vivo, [^15^O]H_2_O positron emission tomography (PET) is considered the reference standard for quantification of absolute MBF owing to the unique characteristics of [^15^O]H_2_O being freely diffusible and completely extracted independent of flow rates [8]. Studies comparing quantitative CMR perfusion with [^15^O] H_2_O PET are however scarce and have been restricted to small sample sizes. Therefore, the aim of the present study was to determine the agreement between CMR and [^15^O]H_2_O PET measurements of absolute MBF and MFR in a relatively large group of patients with stable CAD.

## Material and methods

### Study population and design

Sixty patients with stable CAD referred on a clinical basis to the Amsterdam University Medical Centers, location VUmc, were prospectively enrolled. Exclusion criteria were presence of myocardial scar on late gadolinium enhancement, history of coronary artery bypass grafting, acute myocardial infarction, atrial fibrillation, significant valvular disease, heart failure, non-ischemic cardiomyopathy, renal insufficiency (eGFR < 45 mL/min), and contraindications to intravenous adenosine or CMR. All patients underwent [^15^O]H_2_O PET followed by CMR within 7 days irrespective of the results of [^15^O]H_2_O PET. Revascularization procedures and modifications to pharmacological therapy were not permitted in-between PET and CMR. All study procedures complied with the 1964 Helsinki declaration and its later amendments. The protocol was approved by the Medical Ethics Review Committee of the Amsterdam UMC, location VUmc. Written informed consent was obtained from all individual participants included in the study.

### Positron emission tomography

[^15^O]H_2_O PET was performed using a Gemini TF 64 hybrid PET/CT scanner (Philips Healthcare, Best, the Netherlands). Patients were instructed to refrain from products containing caffeine or xanthine for 24 h prior to scanning. Images were first obtained during resting conditions and thereafter during vasodilator stress. The PET sequence has been described in detail previously [9]. Briefly, 370 MBq of [^15^O]H_2_O was injected intravenously as a 5 mL bolus (0.8 mL/s), immediately followed by a 35 mL saline flush (2 mL/s). A dynamic PET emission scan of 6 min was started simultaneously with tracer administration. After a delay of 15 min, an identical PET sequence was performed during continuous infusion of adenosine through a second venous cannula at a dose of 140 μg/kg/min. Adenosine was started 2 min prior to PET scanning to ensure maximal vasodilation. To correct for photon attenuation and scatter, low-dose (10 mA) respiration-averaged computed tomography scans were obtained during normal breathing just before the rest scan and immediately after the stress scan. Post-processing of PET data was done by a single observer (PvD), who was blinded to all clinical and CMR data. Parametric images of rest and stress perfusion were generated in approximately 10 min using an in-house developed software package, Cardiac VUer [10]. Absolute MBF was quantified in mL per minute per g of perfusable tissue.

### Cardiac magnetic resonance imaging

Again, patients were instructed to refrain from products containing caffeine or xanthine for 24 h prior to image acquisition. All CMR images were obtained on a 1.5-T whole body MR scanner (Magnetom Avanto, Siemens, Erlangen, Germany). Perfusion imaging was performed using a dual sequence, single bolus technique [11], implemented as a Siemens works in progress software by C. Glielmi. Perfusion images were acquired using an echo-planar imaging sequence in three parallel short-axis slices planned at the basal, mid, and apical levels. To assess the arterial input function, low-resolution turboFLASH images were obtained at the basal level using a sequence optimized for the high gadolinium concentration. Perfusion images were obtained every heartbeat for 50–70 cardiac cycles following intravenous injection of a 0.075 mmol/kg bolus of a gadolinium-based contrast agent (DOTAREM®, Guerbet, Villepinte, France). Patients were asked to hold their breath as long as possible and breathe slowly thereafter. In-plane respiratory motion of the heart was corrected using non-rigid registration [12]. Perfusion images were corrected for surface coil-induced signal inhomogeneities using a separate prescan normalization [13]. Typical in-plane resolution of the myocardial perfusion images was 2.5 × 2.5 mm, with a slice thickness of 10 mm (pre-pulse 90°, repetition time 5.6 ms, echo planar factor 4, echo time 1.1 ms, saturation time 110 ms, flip angle 18^°^, matrix size 160 × 144, parallel imaging in the temporal direction [TGRAPPA] [14] factor 2). Perfusion imaging was performed first during vasodilator stress, which was induced by continuous infusion of adenosine using the same protocol as applied during PET. Rest perfusion images were obtained 15 min after stress imaging using identical scanning parameters and slice location. Left ventricular cardiac function was assessed in between stress and rest perfusion with steady-state free-precession cine imaging. Late gadolinium enhancement (LGE) was performed 12–15 min after rest perfusion using a 2D segmented inversion-recovery gradient-echo pulse sequence. Analysis of CMR data was done by a single observer (HE), who was blinded to all clinical and PET data. Post-processing of CMR perfusion images was performed in approximately 15 min using dedicated research software (MASS version 2017-Exp, Leiden, the Netherlands). A region of interest was placed in the LV blood pool of the image series obtained for the arterial input function. Care was taken to avoid inclusion of papillary muscles. Endocardial and epicardial contours were drawn manually on a single phase of each slice of the myocardial perfusion images. Subsequently, these contours were propagated to the other phases. Care was taken to avoid inclusion of blood pool or epicardial fat. Rest and stress MBF were quantified in mL per minute per g using Fermi function-constrained deconvolution, as described previously [15]. Cine and LGE images were analyzed using a commercially available software (QMASS version 7.6, Medis, Leiden, the Netherlands). Left ventricular (LV) end-diastolic volume, end-systolic volume, and ejection fraction were calculated from the cine images. LGE images were visually assessed in order to rule out presence of LV scar tissue.

### Data analysis

Perfusion data were analyzed according to the 17-segment model of the American Heart Association (AHA) [16]. The apical cap (segment 17) was excluded from analysis since this segment was not in the imaging planes of the CMR perfusion acquisition. Myocardial segments were also excluded from analysis if either PET or CMR perfusion imaging was of insufficient quality. Global rest and stress MBF were calculated by averaging perfusion over all 16 segments. In addition, myocardial segments were allocated to the three vascular territories (LAD, left anterior descending; LCx, left circumflex artery; and RCA, right coronary artery) as follows: LAD, segments 1,2,7,8,13,14; LCx, segments 5,6,11,12,16; and RCA, segments 3,4,9,10,15. Rest and stress MBF were calculated for each vascular territory by averaging perfusion over the corresponding myocardial segments. Myocardial flow reserve (MFR) was defined as the ratio of stress to rest MBF and was calculated on a global as well as a regional level. Concordance between CMR and PET was assessed on a per-vessel basis. For [^15^O]H_2_O PET, stress MBF ≤ 2.3 mL/min/g and MFR ≤ 2.5 were considered abnormal according to previously validated cutoff values for diagnosing hemodynamically obstructive CAD (i.e., fractional flow reserve ≤ 0.80) [17]. Receiver operator characteristic (ROC) curve analysis and the Youden index were used to define optimal cutoff values for CMR measurements of stress MBF and MFR (MBF_CMR_ and MFR_CMR_).

### Statistical analysis

Continuous variables are presented as mean ± standard deviation or median with inter-quartile range. Categorical variables are expressed as frequency with percentage. Pearson’s correlation was used to quantify association between continuous variables. Agreement between PET and CMR perfusion was assessed by intraclass correlation coefficients (ICCs) and visually by Bland-Altman analysis. ICCs for absolute agreement of single measures were estimated using a two-way mixed effects model. Paired samples’ T-tests were used to compare the means in heart rate and global perfusion measurements between CMR and PET. To account for clustering of multiple vessel measurements per patient, means of regional perfusion indexes were compared using a mixed linear model with a fixed effect for imaging technique and random effects for patient and vessel nested within patient. All statistical tests were two tailed, and a *p* value of <0.05 was considered statistically significant. Statistical analysis was done with SPSS (version 22 for Windows, IBM, Armonk, New York, United States of America).

## Results

**Fig. 1:**
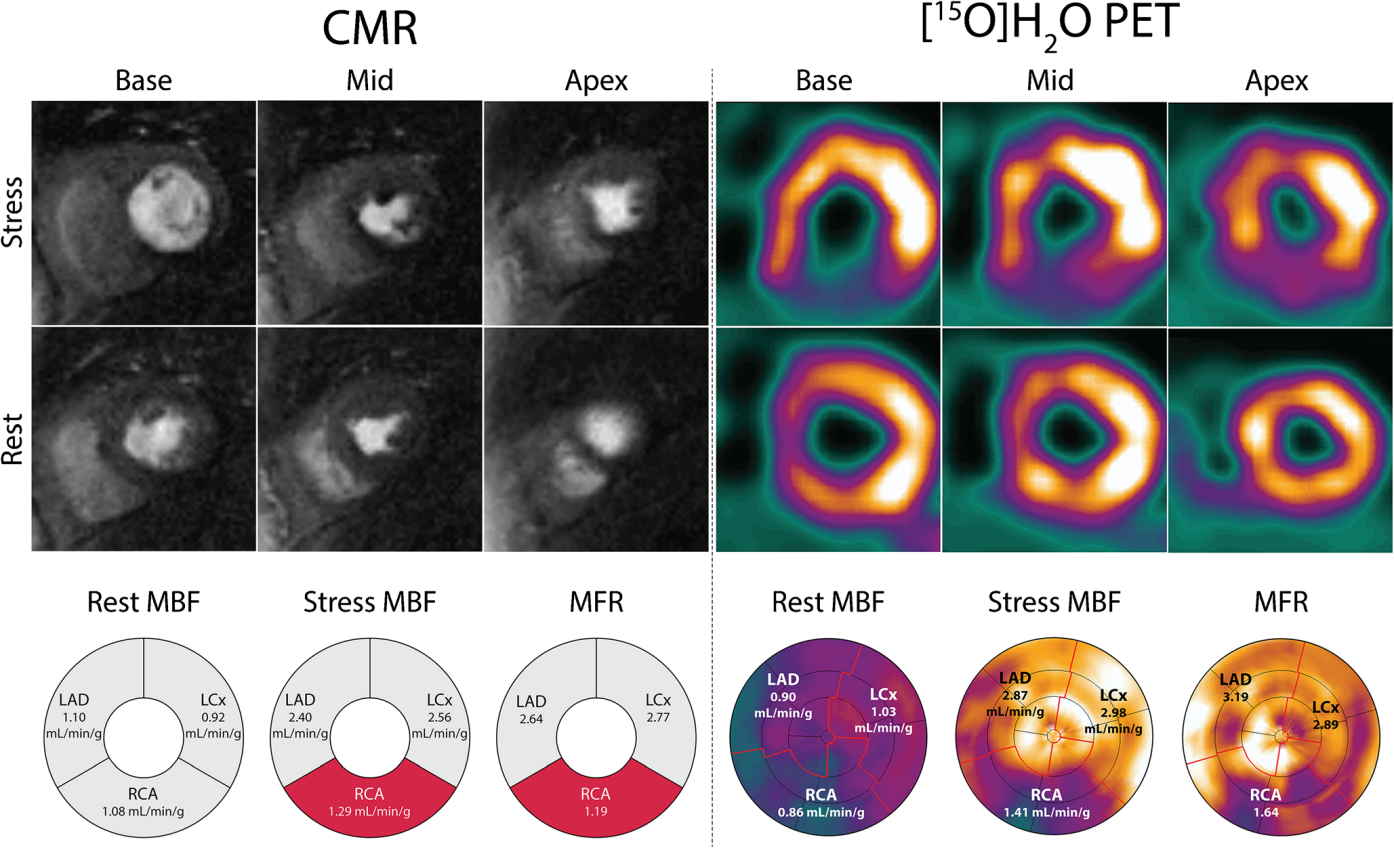
Case example of concordance between CMR and [^15^O]H_2_O PET in a 71-year-old female patient who presented with typical angina. Short-axis slices at the basal, mid, and apical levels have been selected from the PET study in order to match CMR and PET images. Both CMR and [^15^O]H_2_O PET demonstrate a perfusion defect in the inferior wall stretching from base to apex. With both techniques, the measured stress MBF and MFR in the vascular territory of the RCA are well below the ischemic thresholds. CMR = cardiac magnetic resonance imaging; LAD = left anterior descending artery; LCx = left circumflex artery; MBF = myocardial blood flow; MFR = myocardial flow reserve; PET = positron emission tomography; RCA = right coronary artery

[^15^O]H_2_O PET was successfully performed in all patients. Stress perfusion CMR images were deemed of insufficient quality in one (2%) patient, which was excluded from analysis. In an additional three (5%) patients, rest perfusion imaging was omitted from the CMR scanning protocol. Baseline characteristics of the final cohort of 59 patients are shown in Table 1. Median time between PET and CMR was 5 [5] days. Table 2 lists data on CMR-derived LV function and volumes. LV ejection fraction was normal, with a mean of 63 ± 5%. Resting heart rate during perfusion imaging did not differ between CMR and PET (63 ± 9 vs. 64 ± 11 bpm; *P* = 0.52). Heart rate during vasodilator stress (89 ± 14 vs. 87 ± 14 bpm; *P* = 0.21) and the increment in heart rate (26 ± 12 vs. 22 ± 12 bpm; *P* = 0.11) was also similar for both techniques, indicating equal hemodynamic response to adenosine. Figure 1 shows a case example of concordance between CMR and PET in a patient with severely impaired stress perfusion and MFR in the vascular territory of the RCA.

**Table 1:** Baseline characteristics of the patient cohort

Variables	
No. of patients	59
Age (years)	63 ± 9
Male gender	41 (70%)
Body mass index (kg/m^2^)	27 ± 4
Risk factors	
Family history of CAD	31 (53%)
Hypertension	40 (68%)
Dyslipidemia	41 (70%)
Diabetes mellitus	5 (9%)
Smoking	28 (48%)
Medication	
ACE inhibitor or ATII antagonist	23 (39%)
Aspirin	58 (98%)
Beta-blocker	33 (56%)
Calcium channel blockers	18 (30%)
Long-acting nitrates	18 (30%)
Statin	50 (85%)
Symptoms	
Asymptomatic	3 (5%)
Dyspnea	14 (24%)
Non-anginal chest pain	5 (9%)
Atypical angina	17 (29%)
Typical angina	20 (34%)

Data are mean ± standard deviation or absolute number (%). *ACE* , angiotensin-converting-enzyme; *ATII* ,angiotensin II receptor; *CAD* ,coronary artery disease

**Table 2:** CMR-derived LV volumes and function

Variables	
Left ventricular end-systolic volume (mL)	59 ± 19
Left ventricular end-diastolic volume (mL)	158 ± 36
Left ventricular ejection fraction (%)	63 ± 5
Left ventricular mass (g)	92 ± 28

Data are mean ± standard deviation

### Global myocardial perfusion

**Fig. 2:**
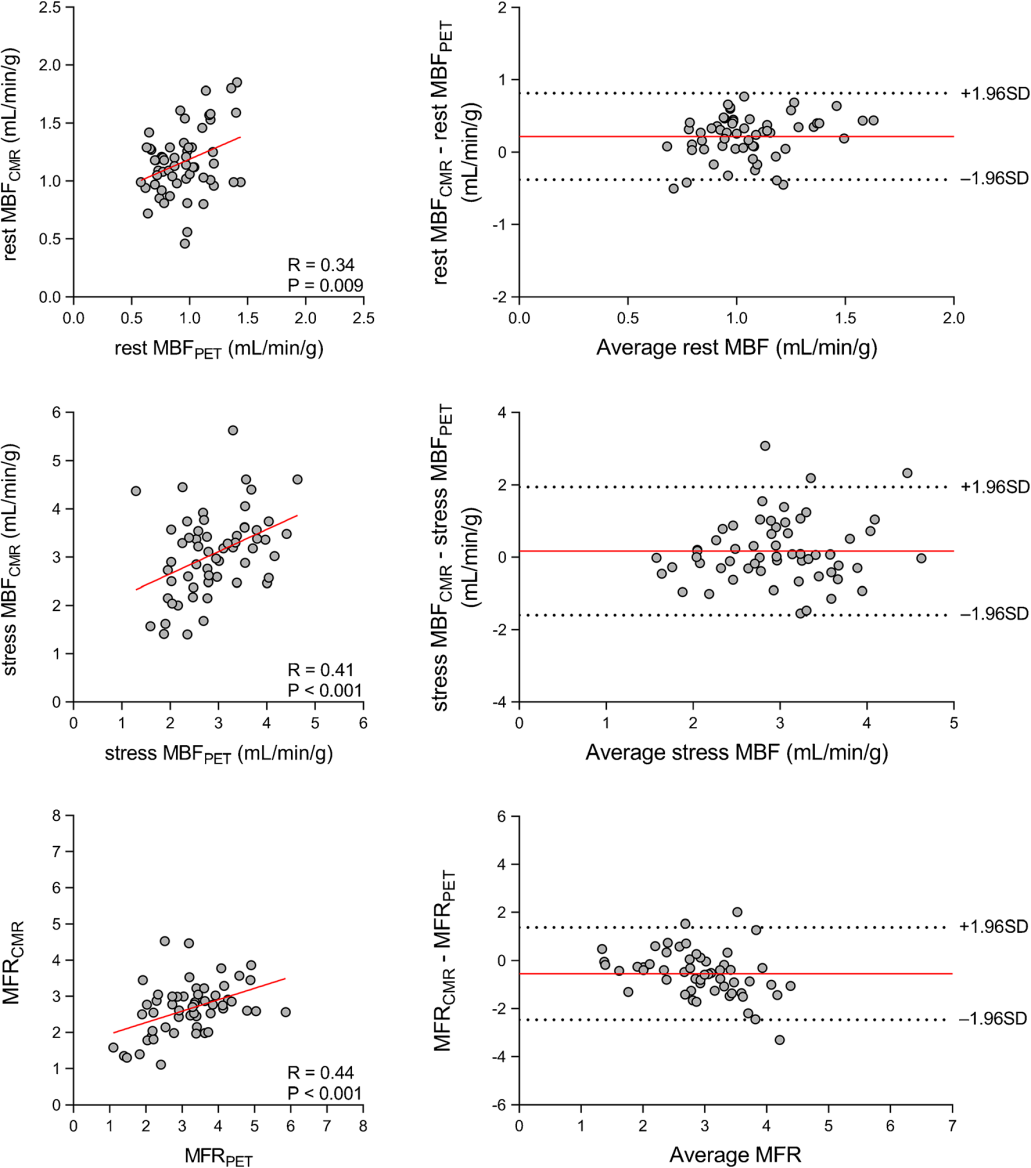
Global perfusion. Scatter (**left**) and Bland-Altman (**right**) plots demonstrating the relationship between CMR and [^15^O]H_2_O PET measurements of global rest MBF (**top**) and stress MBF (**middle**) and MFR (**bottom**). In the Bland-Altman plots, the solid red line indicates the mean bias, and the dashed black lines indicate the limits of agreement. Abbreviations as in Fig. 1

Figure 2 displays the relationship between CMR and PET measurements of global myocardial perfusion. CMR-derived MBF (MBF_CMR_) and PET-derived MBF (MBF_PET_) obtained during vasodilator stress showed only moderate correlation (*r* = 0.41; *P* < 0.001) and poor to moderate inter-method reliability (ICC for absolute agreement = 0.40 [95% confidence interval (CI): 0.17 to 0.59]; *P* < 0.001). In addition, CMR-derived MFR (MFR_CMR_) and PET-derived MFR (MFR_PET_) showed a moderate correlation (*r* = 0.44; *P* < 0.001) and poor to moderate inter-method reliability (ICC for absolute agreement = 0.36 [95% CI: 0.09 to 0.58]; *P* < 0.001). Bland-Altman analysis demonstrated a mean bias of 0.2 ± 0.9 mL/min/g for stress MBF and − 0.5 ± 1.0 for MFR. Table 3 (top row) displays the mean values of global rest MBF, stress MBF and MFR as measured using CMR and PET. CMR measurements of global rest MBF were significantly higher than those obtained using PET (1.2 ± 0.3 vs. 0.9 ± 0.2 mL/min/g; *P* < 0.001), whereas global stress MBF did not differ between the techniques (3.1 ± 0.9 vs. 2.9 ± 0.8 mL/min/g; *P* = 0.14). Global MFR was significantly lower for CMR in comparison to PET (2.6 ± 0.7 vs. 3.2 ± 1.0; *P* < 0.001).

**Table 3:** Means of CMR and PET measurements of absolute MBF and MFR

Variables	CMR	[^15^O]H_2_O PET	P value
*Global perfusion*			
Rest MBF (mL/min/g)	1.2 ± 0.3	0.9 ± 0.2	< 0.001
Stress MBF (mL/min/g)	3.1 ± 0.9	2.9 ± 0.8	0.14
MFR	2.6 ± 0.7	3.2 ± 1.0	< 0.001
*Regional perfusion*			
Rest MBF (mL/min/g)	1.2 ± 0.3	0.9 ± 0.2	< 0.001
Stress MBF (mL/min/g)	3.1 ± 0.9	2.9 ± 0.8	0.014
MFR	2.7 ± 0.9	3.2 ± 1.1	< 0.001

Data are mean ± standard deviation. *CMR* ,cardiac magnetic resonance imaging; *MBF* ,myocardial blood flow; *MFR* ,myocardial flow reserve; *PET* ,positron emission tomography

### Regional myocardial perfusion

**Fig. 3:**
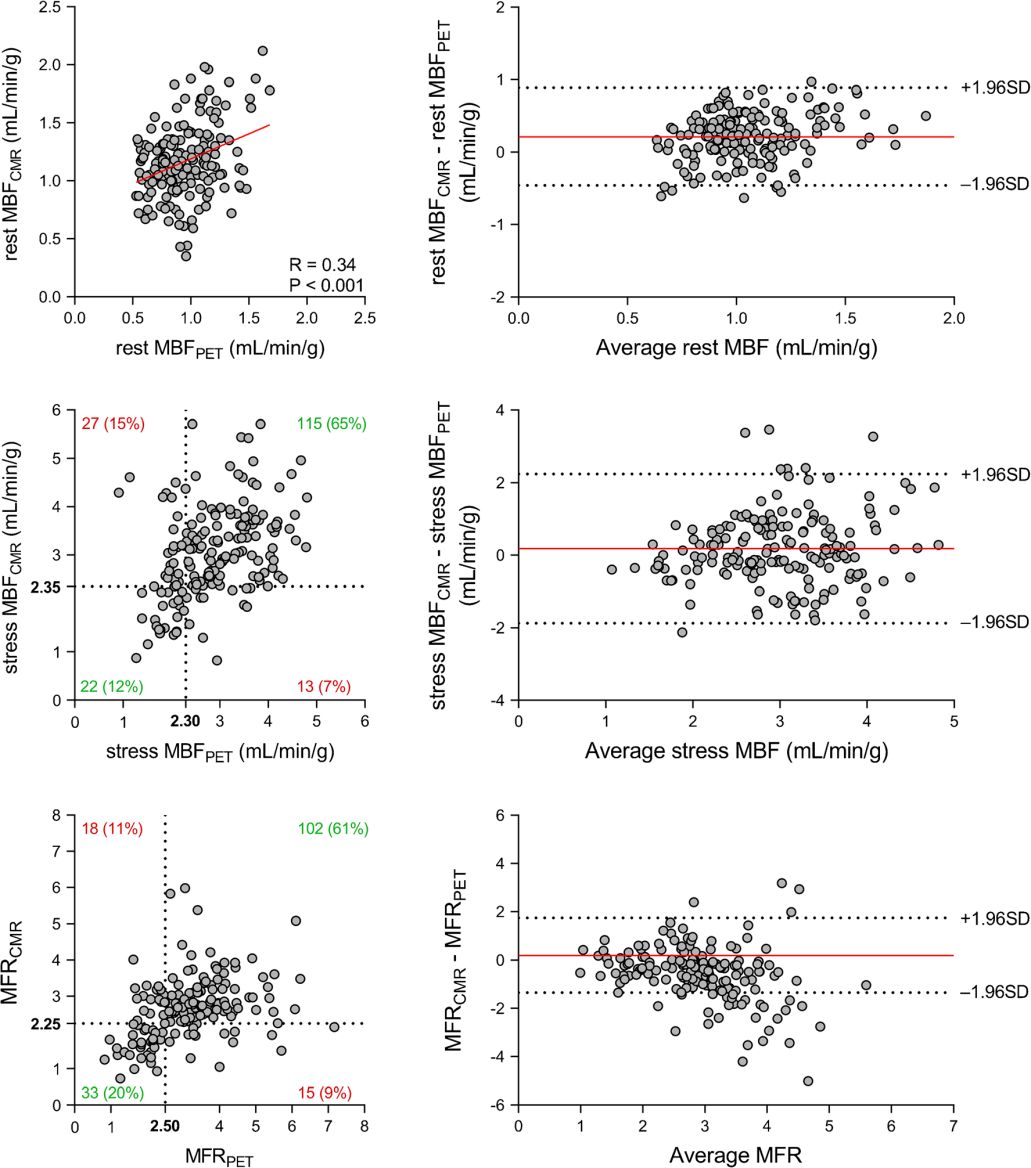
Regional perfusion. Scatter (**left**) and Bland-Altman (**right**) plots comparing CMR and [^15^O]H_2_O PET measurements of rest MBF (**top**) and stress MBF (**middle**) and MFR (**bottom**) on a per-vessel basis. In the scatter plots of stress MBF and MFR, the dashed black lines indicate the thresholds for abnormal myocardial perfusion. Stress MBF and MFR correlate significantly between CMR and [^15^O]H_2_O PET (*r* = 0.39; *P* < 0.001 for stress MBF and *r* = 0.36; *P* < 0.001 for MFR). In the Bland-Altman plots, the solid red line indicates the mean bias, and the dashed black lines indicate the limits of agreement. Abbreviations as in Fig. 1

**Fig. 4:**
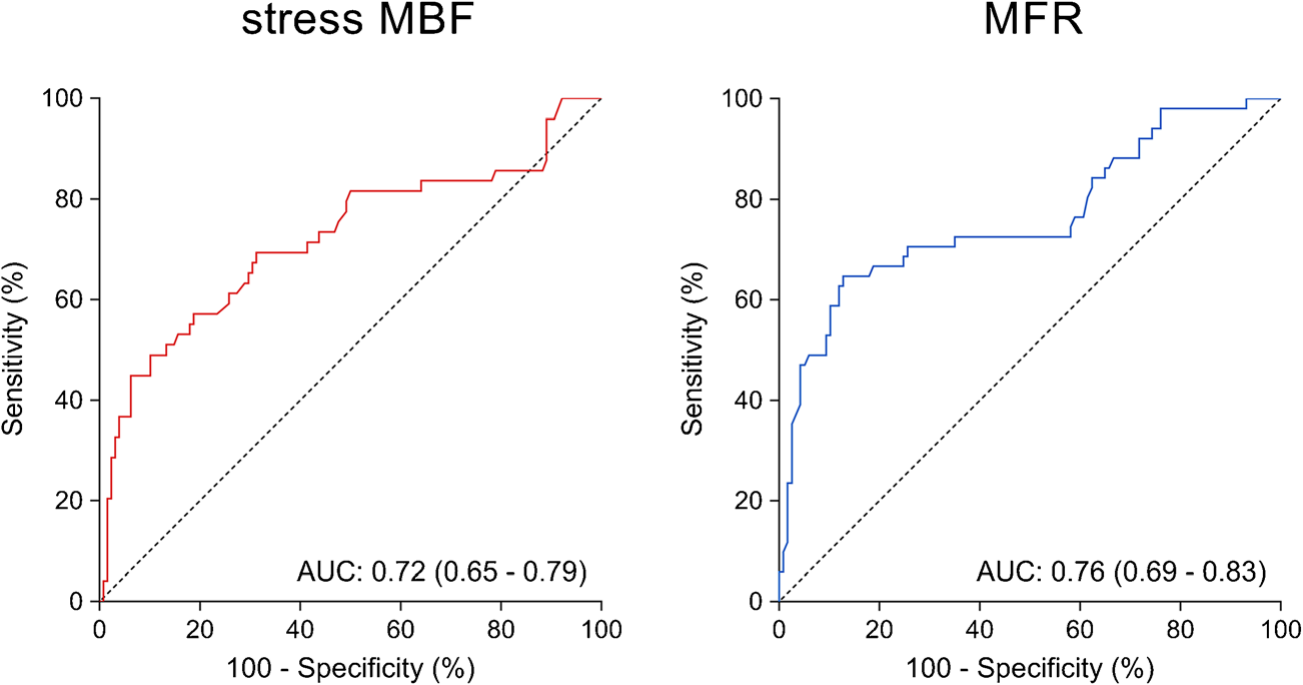
ROC curves for detecting abnormal regional perfusion. ROC curves of CMR derived stress MBF (**left**) and MFR (**right**) for detecting abnormal regional perfusion defined as [^15^O]H_2_O PET-derived stress MBF ≤2.30 and MFR ≤2.50. AUC = area under the curve; ROC = receiver operating characteristic; other abbreviations as in Fig. 1

The relationship between CMR and PET measurements of regional myocardial perfusion is shown in Fig. 3. On a per vessel basis, stress MBF_CMR_ and stress MBF_PET_ showed only moderate correlation (*r* = 0.39; *P* < 0.001) and poor inter-method reliability (ICC for absolute agreement = 0.38 [95% CI: 0.25 to 0.50]; *P* < 0.001). In addition, only modest correlation (*r* = 0.36; *P* < 0.001) and poor inter-method reliability (ICC for absolute agreement = 0.30 [95% CI: 0.13 to 0.46]; *P* < 0.001) were present between MFR_CMR_ and MFR_PET_. Bland-Altman analysis revealed a mean bias of 0.2 ± 1.0 for stress MBF and − 0.5 ± 1.2 for MFR. CMR demonstrated a tendency to underestimate MFR at higher values. Table 3 (bottom rows) lists the mean values of CMR and PET measurements of rest MBF and stress MBF and MFR. Rest and stress MBF were significantly higher for CMR compared with PET (1.2 ± 0.3 vs. 0.9 ± 0.2 mL/min/g; *P* < 0.001 for rest MBF and 3.1 ± 0.9 vs. 2.9 ± 0.8 mL/min/g; *P* = 0.014 for stress MBF). Conversely, MFR_CMR_ was significantly lower than MFR_PET_ (2.7 ± 0.9 vs. 3.2 ± 1.1; *P* < 0.001). [^15^O]H_2_O PET demonstrated abnormal stress MBF and MFR in respectively 49 (27%) and 53 (29%) vascular territories. Figure 4 displays the ROC curves of quantitative CMR perfusion imaging for detecting abnormal stress MBF and MFR as defined by [^15^O]H_2_O PET. Stress MBF_CMR_ displayed an area under the curve (AUC) of 0.72 (95% CI: 0.65 to 0.79) and had an optimal cutoff value of 2.35 mL/min/g. MFR_CMR_ had an AUC of 0.76 (95% CI: 0.69 to 0.83) and an optimal cutoff value of 2.25. Using these cutoff values, stress MBF_CMR_ and MFR_CMR_ were abnormal in respectively 35 (20%) and 48 (29%) vascular territories. CMR and PET were concordant in 137 (77%) vascular territories for stress MBF and in 135 (80%) vascular territories for MFR.

## Discussion

The present study is the largest to date investigating the agreement between CMR and PET measurements of absolute myocardial perfusion. State-of-the-art methods were applied for both CMR and PET perfusion imaging. [^15^O]H_2_O, the gold standard for quantification of absolute MBF, was used as tracer for PET, and a dual sequence, single bolus technique optimized for quantification of absolute MBF was used for CMR perfusion imaging. The main finding is that CMR and [^15^O]H_2_O PET measurements of stress MBF and MFR showed only modest agreement but were nevertheless concordant in 77% of vascular territories for stress MBF and in 80% of vascular territories for MFR.

Previous – predominantly PET – studies have shown quantification of MBF to improve both prognostic and diagnostic performance in the management of patients with CAD [2, 4, 18–20]. With regard to detection of obstructive CAD, quantitative perfusion measures have been shown to be particularly useful in unmasking balanced ischemia due to three-vessel or left main disease and increase conspicuity of subtle (subendocardial) ischemia [21]. In addition, absolute stress MBF and MFR may also provide insight in coronary microvascular function [22]. Although cardiac PET is the commonly used tool for quantitative perfusion imaging, CMR has gained increasing interest for MBF imaging because of its wide availability, high spatial resolution, and non-ionizing nature. In addition, it may also provide information on left ventricular function and viability rendering CMR ideally suited for the noninvasive assessment of CAD.

Previous studies comparing quantitative CMR and PET perfusion have been limited to small numbers of subjects and differ markedly in study population (i.e., patients with CAD vs. healthy volunteers), tracer used for PET quantification, CMR acquisition technique, and CMR field strength. Pärkkä et al. performed CMR and [^15^O]H_2_O PET in 18 healthy volunteers and reported a significant correlation between CMR and PET measurements of stress MBF (*r* = 0.70) and MFR (*r* = 0.46), although MFR_CMR_ was found to be lower than MFR_PET_ [23]. Fritz-Hansen et al. and Pack et al., who performed CMR perfusion imaging and ^13^N-ammonia PET in 10 and 4 healthy volunteers, respectively, reported similar results [24, 25]. In contrast, Tomiyama et al. studied 10 healthy volunteers with [^15^O]H_2_O PET and CMR perfusion imaging at 3-T and documented a strong correlation (*r* = 0.83) between regional values of MFR_CMR_ and MFR_PET_ [26]. The agreement between quantitative CMR and PET perfusion in patients with CAD was studied by Qayyum and colleagues [27]. Fourteen patients underwent rubidium-82 PET followed by CMR. Regional MFR, quantified with CMR using a single sequence, single bolus technique, was found to correlate well with PET-derived flow reserve (*r* = 0.82). Morton et al. used a dual bolus technique to investigate the agreement between quantitative CMR and PET perfusion in patients with CAD [28]. CMR measurements of rest and stress MBF showed modest correlation with PET-derived values (*r* = 0.32 and *r* = 0.37), yet MFR_CMR_ correlated strongly with MFR_PET_ (*r* = 0.79). Importantly, CMR and PET displayed equal diagnostic performance in a head-to-head comparison against invasive coronary angiography, indicating that although the correlation between CMR and PET in terms of absolute MBF values is modest, diagnostic performance appears to be non-inferior to PET. Engblom et al. and Kunze et al. employed a dual sequence, single bolus technique to quantify absolute MBF with CMR, avoiding multiple contrast bolus injections while preserving accurate caption of the arterial input function [11, 29, 30]. CMR and ^13^N-ammonia PET were performed in respectively 21 and 29 patients with stable CAD, and pooled rest and stress measurements of regional MBF were found to correlate strongly between the techniques (*r* = 0.83 and *r*^2^ = 0.72). Finally, Kero et al. recently performed CMR and [^15^O]H_2_O PET in 15 patients with stable CAD using a single sequence, single bolus CMR technique [31]. Although regional values of stress MBF showed moderate correlation between CMR and PET (*r* = 0.69), MFR_CMR_ correlated poorly with MFR_PET_ (*r* = 0.08). Notwithstanding the interesting results of this study, the small sample size and potential inclusion of patients with myocardial scar may have influenced their findings. In addition, the single sequence single bolus technique used is considered suboptimal for quantification of absolute MBF [32].

The results of the present study corroborate these prior reports, as we demonstrate only modest correlation between quantitative CMR and PET perfusion measurements. Inter-method reliability between CMR and PET is poor to moderate, as ICC values range from 0.30 to 0.40 with upper bounds of the 95% confidence interval not exceeding 0.60. Although the Bland-Altman plots demonstrate a small mean bias, the limits of agreement are wide, meaning that substantial differences between CMR and PET measurements of stress MBF and MFR are present. It is important to realize however that although [^15^O]H_2_O PET is not affected by the “roll-off phenomenon,” which occurs with all other myocardial perfusion tracers, the range of perfusion that is clinically important lies apparently beneath this threshold [33]. This may explain why, despite the modest agreement, stress MBF and MFR are concordant between CMR and PET in the majority of vascular territories. Further support to this hypothesis is provided by a recent meta-analysis reporting a high diagnostic accuracy of quantitative CMR perfusion [34]. We also observed significantly higher values of rest and stress MBF for CMR compared with PET, which may have resulted from underestimation of the arterial input function with CMR. Although the current dual sequence approach is designed to preserve linearity between gadolinium concentration and signal intensity in the blood pool, saturation effects due to T2* decay still significantly impact the arterial input curve [35]. Similar to previous reports, we also found that MFR_CMR_ is lower than MFR_PET_, particularly at higher values. The main reason for this lies in the kinetic properties of gadolinium-based contrast agents. The extraction fraction of gadolinium is approximately 0.55 at rest and decreases unpredictably with increasing flow rates [36]. This results in an underestimation of the tissue response curves at higher flows, subsequently leading to an underestimation of MFR.

### Study limitations

The present study lacks invasive confirmation of hemodynamically obstructive CAD. Although [^15^O]H_2_O PET is considered to be the reference standard for quantification of myocardial perfusion, invasive measurements of fractional flow reserve are the preferred reference for diagnosing hemodynamically obstructive CAD and guiding revascularization. Therefore, our results urge for a new study comparing quantitative PET and CMR head-to-head against invasive measures of physiology. Secondly, resting flow is known to vary according to metabolic demand [37]. Some of the observed discrepancy between CMR and PET is therefore not attributable to differences in methodology but results from physiological fluctuations in rest flow. Finally, the sequence of imaging was similar in all patients as [^15^O]H_2_O PET was always performed prior to CMR with a maximum delay of 7 days. Although medication and treatment were kept constant, physiological changes in myocardial perfusion may have occurred in-between PET to CMR.

## Conclusions

CMR measurements of rest MBF, stress MBF and MFR showed only modest agreement to those obtained with [^15^O]H_2_O PET. Nevertheless, stress MBF and MFR were concordant between CMR and [^15^O]H_2_O PET in 77% and 80% of vascular territories, respectively.
